# Performance Evaluation of AFIAS ST2 and Ichroma ST2 Assays in Comparison with Presage ST2 Assay

**DOI:** 10.31083/j.rcm2404100

**Published:** 2023-03-31

**Authors:** Hanah Kim, Tae-Hwan Lee, Mina Hur, Hyun-Joong Kim, Hyun Suk Yang, Kyeong Ryong Lee, Salvatore Di Somma

**Affiliations:** ^1^Department of Laboratory Medicine, Konkuk University School of Medicine, 05030 Seoul, Republic of Korea; ^2^Division of Cardiology, Department of Internal Medicine, Konkuk University School of Medicine, 05030 Seoul, Republic of Korea; ^3^Department of Emergency Medicine, Konkuk University School of Medicine, 05030 Seoul, Republic of Korea; ^4^Department of Medical-Surgery Sciences and Translational Medicine, School of Medicine and Psychology, Sapienza–University, Sant' Andrea Hospital, 00189 Rome, Italy

**Keywords:** soluble suppression of tumorigenicity 2, point-of-care, performance, cut-off, comparison, prognosis, heart failure

## Abstract

**Background:**

Elevated soluble suppression of tumorigenicity 2 (sST2) levels may predict mortality in heart failure (HF) patients. The AFIAS ST2 assay (AFIAS ST2, Boditech Med Inc., Chuncheon, Korea) and ichroma ST2 assay (ichroma ST2, Boditech Med Inc.) are newly developed point-of-care (POC) assays for measuring sST2 level. We evaluated the performance of these assays, in terms of cut-off validation and prognosis, and compared them with that of the Presage ST2 assay (Presage ST2, Critical Diagnostics, San Diego, CA, USA).

**Methods:**

We validated the US FDA-claimed sST2 clinical cut-off of 35 ng/mL using 420 serum samples (298 samples from the universal sample bank of the American Association for Clinical Chemistry and 122 samples from reference individuals from Konkuk University Medical Center). We compared AFIAS ST2 and ichroma ST2 with Presage ST2, using 206 samples from patients with HF. We assessed prognosis using the three assays in 252 samples from the Barcelona ambulatory HF cohort subsets.

**Results:**

The upper reference limits of AFIAS ST2 and ichroma ST2 were within the clinical cut-off of Presage ST2. The results of AFIAS ST2 and ichroma ST2 were highly correlated with those of Presage ST2 (r = 0.82 and 0.81, respectively). Based on this cut-off, all three assays predicted cardiovascular death.

**Conclusions:**

The new POC assays, AFIAS ST2 and ichroma ST2, would be useful in clinical practice for managing HF patients, with performances equivalent to that of Presage ST2.

## 1. Introduction

The American College of Cardiology/American Heart Association (ACC/AHA) defines stage B heart failure (HF), or pre-HF, as a structural heart disease without current or previous symptoms and (or) signs of HF [1]. Patients with structural heart dysfunction, who do not exhibit any clinical symptoms, are usually not diagnosed unless they undergo imaging [2]. Echocardiography is essential for diagnosing HF with reduced ejection fraction; however, it requires expensive equipment, a well-trained physician, and operating time for screening asymptomatic individuals [1, 2]. Therefore, early detection of patients with stage B HF can be challenging in clinical practice.

B-type natriuretic peptide (BNP) and its N-terminal (NT)-prohormone BNP (NT-proBNP) are released in response to changes in pressure inside the heart. Both BNP and NT-proBNP levels have been used for risk stratification and disease monitoring in acute and chronic HF [1, 2, 3, 4, 5, 6]. These levels can be measured using high-throughput automated immunoassays or point-of-care (POC) assays [7]. However, they may be affected by noncardiac factors, such as age, anemia, and kidney diseases [1, 8]. Therefore, clinicians should be careful when interpreting their results [1].

Suppression of tumorigenicity 2 (ST2) belongs to the interleukin (IL)-1 receptor family and exists in two forms: a ligand isoform (ST2L) and soluble isoform (sST2) [8]. In cardiac myocytes, binding of IL-33 to ST2L has a cardioprotective effect [5]. However, if it binds to sST2, the protective effect decreases. This indicates that elevated sST2 levels are associated with the prognosis of acute or chronic HF [5, 8]. Compared with other cardiac markers, such as cardiac troponin and NT-proBNP, sST2 is known to be less influenced by non-cardiac factors and more specific to HF [5, 8, 9, 10]. Several sST2 assays are commercially available. The Human ST2/IL-33R DuoSet (R&D Systems, Minneapolis, MN, USA) is a research use only (RUO) assay. The Aspect-PLUS ST2 Rapid Test (Aspect-PLUS ST2, Critical Diagnostics, San Diego, CA, USA), Sequent-IA ST2 assay (Sequent-IA ST2, Critical Diagnostics), and Presage ST2 assay (Presage ST2, Critical Diagnostics) are *in vitro* diagnostic (IVD) assays [11, 12, 13, 14]. Among them, Presage ST2 is the only US Food and Drug Administration (FDA)-approved assay for clinical use that can accurately measure low circulating sST2 levels in healthy individuals; a clinical cut-off of 35 ng/mL is used based on the HF: A Controlled Trial Investigating Outcomes of Exercise Training (HF-ACTION) study [15, 16].

Presage ST2 is an enzyme-linked immunosorbent assay (ELISA), and POC assays would be more practical for the timely diagnosis and swift treatment of HF patients [10, 17, 18]. Aspect-PLUS ST2 has been compared with Presage ST2 in terms of analytical performance and prognosis prediction; however, the US FDA-claimed clinical cut-off has not been validated using reference individuals [13]. The AFIAS ST2 assay (AFIAS ST2, Boditech Med Inc., Chuncheon, Korea) and ichroma ST2 assay (ichroma ST2, Boditech Med Inc.) are newly developed IVD POC assays for measuring sST2 levels. In this study, we evaluated and compared the performance of these two assays with that of the conventional Presage ST2 assay. For all three sST2 assays, we validated the clinical cut-off, compared their analytical performances, and assessed their equivalence for predicting prognosis. All evaluations were performed according to the Clinical and Laboratory Standards Institute (CLSI) guidelines.

## 2. Materials and Methods

### 2.1 Study Population

This *in vitro *experimental study was conducted at Konkuk University Medical Center (KUMC), Seoul, Korea, from July 2020 to January 2021. The study protocol was designed in accordance with the Declaration of Helsinki and was approved by the Institutional Review Board of KUMC. This study used anonymized samples and required neither additional sampling nor therapeutic intervention. Therefore, the requirement of obtaining written informed consent from the enrolled individuals was waived.

The study population consisted of four subsets: samples from the universal sample bank of the American Association for Clinical Chemistry (AACC) [19], samples from KUMC healthy individuals, samples from KUMC HF patients, and samples from the Barcelona ambulatory HF cohort subsets (Barcelona samples) [20]. The AACC and KUMC healthy individual samples were used for cut-off validation, the samples from KUMC HF patients for assay comparison, and the Barcelona samples for the equivalence of prognosis prediction. The AACC samples were a part of the full sample set (n = 800) that the assay manufacturer (Boditech Med Inc.) purchased from the AACC sample bank (invoice #1084290) with a purchase request (inclusion criteria: mixed males and females in all-age ranges). After the internal use for assay development and validation, the manufacturer provided remaining samples randomly with associated information. The manufacturer also purchased 300 samples (invoice # FV20/0035) from the Barcelona ambulatory HF cohort subsets. After excluding 48 samples obtained from patients who died for reasons other than cardiovascular (CV) events, the manufacturer provided the remaining 252 samples with associated information. For the KUMC samples (n = 123 from healthy individuals and n = 206 from patients with HF), we used samples that were leftover after routine laboratory testing. The characteristics of the study population are summarized in Table 1 (Ref. [19, 20]).

**Table 1.** **Characteristics of the study population**.

		Validation of clinical cut-off		Comparison of assays	Equivalence of prognosis prediction
		AACC sample bank (n = 298) [19]	KUMC healthy individuals (n = 122)	KUMC HF samples (n = 206)	Barcelona ambulatory HF cohort subset (n = 252) [20]
Demographics					
	Age, yrs	39 (30–52)	39 (33–49)	64 (57–75)	68 (58–75)
	Male	169 (56)	70 (57)	122 (53)	228 (76)
	Height, cm	NA	NA	NA	165 (158–171)
	Weight, kg	NA	NA	NA	74.8 (63.5–83.3)
	Smoking history	NA	NA	NA	57 (22.6)
	Race	Black, Caucasian, Hispanic, and Asian	Korean	Korean	Black and Caucasian
Clinical variables					
	Diabetes	0	0	NA	123 (48.8)
	Hypertension	0	0	NA	165 (65.5)
	LVEF, %	NA	NA	NA	31 (25–37)
	NYHA class ≥3	NA	NA	NA	48 (19.0)
	Arrhythmia	0	0	NA	31 (12.3)
	CV death	NA	NA	NA	65 (25.8)
	Follow-up duration, yrs	NA	NA	NA	3.6 (2.6–5.0)
Laboratory variables					
	Creatinine	0.9 (0.8–1.0)	0.7 (0.7–0.8)	1.0 (0.8–1.3)*	1.2 (0.9–1.7)
	HbA1c, %	5.6 (5.3–5.8)	NA	NA	NA
	HDL-C, mg/dL	NA	74 (69–85)	NA	NA
	LDL-C, mg/dL	NA	87 (75–94)	NA	NA
	Triglyceride, mg/dL	NA	56 (45–69)	NA	NA
	NT-proBNP, pg/mL	37 (20–78)	NA	1623 (684–8305)*	1910 (881–4240)

Data are presented as numbers (percentages) or medians (IQR). *Data were obtained only from patients who were available at the time. Abbreviations: AACC, American Association of Clinical Chemistry; CV, cardiovascular; HbA1c, hemoglobin A1c; HDL-C, high-density lipoprotein cholesterol; HF, heart failure; KUMC, Konkuk University Medical Center; LDL-C, low-density lipoprotein cholesterol; LVEF, left ventricular ejection fraction; n, number; NA, not available; NT-proBNP, N-terminal prohormone B-type natriuretic peptide; NYHA, New York Heart Association; yrs, years.

### 2.2 Measurement of sST2 Levels

The samples were stored at –70 °C and thawed at 37 °C for measuring sST2 levels. The sST2 levels measured using Presage ST2 were considered as reference, and those measured using AFIAS ST2 and ichroma ST2 were compared with the reference.

Presage ST2 is an ELISA comprising a ready-to-use 96-well microtiter plate coated with mouse monoclonal anti-human sST2 antibodies; spectrophotometric absorbance is measured at 450 nm with a microtiter well reader. The assay was performed using a Gemini automated microplate processor (Stratec Biomedical Systems, Birkenfeld, Germany) [11, 12]. It takes 3 h to measure sST2 levels using Presage ST2, and its hands-on time is 30 min. The measurable range of Presage ST2 was 3.1–200 ng/mL, and its coefficient of variation (%) was less than 10.0%. The limit of detection (LoD) and limit of quantification (LoQ) were 1.8 ng/mL and 2.4 ng/mL, respectively.

AFIAS ST2 is a fluorescent sandwich immunoassay for the automatic quantitative determination of the sST2 antigen. AFIAS ST2 can measure sST2 levels in various samples, such as whole blood, serum, and plasma collected in lithium heparin or EDTA vacutainers. For this study, 100 μL of serum was dispensed into the sample well of the cartridge containing the test strip. After loading the cartridge into the AFIAS-6 system (Boditech Med Inc.), all procedures, from loading the detection buffer into the cartridge to obtaining test results, were automated. Briefly, a fluorescence-labeled antibody conjugate in the detection buffer binds to the antigen in the sample to form antibody-antigen complexes. The complexes migrate onto a nitrocellulose membrane and are captured by antibodies on the test line of the strip. More antigens in the sample form more antigen-antibody complexes, leading to stronger fluorescence intensity [21]. It takes 12 min to obtain sST2 levels using AFIAS ST2, without hands-on time. The manufacturer-claimed measurable range was 3.1–200 ng/mL, and its coefficient of variation (%) was less than 5.0%. The LoD and LoQ were 2.8 ng/mL and 3.1 ng/mL, respectively.

The ichroma ST2 is a manual-type assay with the same principle as that of AFIAS ST2. It can also be used to measure sST2 levels in various samples, such as whole blood, serum, and plasma collected in lithium heparin or EDTA vacutainers. For this study, 150 μL of diluent and 75 μL of serum mixtures were transferred to a detector tube containing a fluorescence-labeled antibody conjugate, and 75 μL of the mixture was loaded into the ichroma cartridge manually. After 12 min, the test results were displayed on the screen of the ichroma II reader (Boditech Med Inc.). It takes 12 min to obtain sST2 levels using ichroma ST2, and its hands-on time is one minute. The manufacturer-claimed measurable range was 3.1–200 ng/mL, and its coefficient of variation (%) was less than 5.4%. The LoD and LoQ were 2.8 ng/mL and 3.1 ng/mL, respectively.

### 2.3 Statistical Analysis

On performing the Shapiro–Wilk test, all data exhibited a non-parametric distribution [22]. Therefore, the data are expressed as medians with interquartile ranges (IQR) for the continuous variables and as numbers with proportions for the categorical and binary variables. Using the Reed method and generalized extreme studentized deviate technique, three outliers were identified and excluded from the analysis (n = 1 in KUMC healthy individuals; n = 2 in KUMC HF patients) [23, 24].

We validated the clinical cut-off (35 ng/mL) according to the CLSI guideline EP28-A3C [25]. The 95th percentile upper reference limit (URL) with a 90% confidence interval (CI) was calculated for the AFIAS ST2 and ichroma ST2 results.

We compared the results of the three assays using Passing–Bablok regression and Bland–Altman plots, according to the CLSI guideline EP09C-ED3 [24]. The correlation coefficients (r) were interpreted as follows: <0.30, negligible; 0.30–0.49, low; 0.50–0.69, moderate; 0.70–0.89, high; and ≥0.90, very high correlations [26]. On the Bland–Altman plot, the mean difference and ±1.96 standard deviations (SD) were interpreted informally to visualize the mean difference. Weighted kappa (κ) values with 95% CI were used to calculate the degree of agreement using the clinical cut-off and were interpreted as follows: <0.20, poor; 0.21–0.40, fair; 0.41–0.60, moderate; 0.61–0.80, good; and >0.81, very good [27].

We assessed the assay equivalence for predicting CV death at a cut-off of 35 ng/mL using areas under the curve (AUC) in receiver operating characteristic (ROC) curves, according to the CLSI guideline EP24-A2 [28]. The sensitivity, specificity, Youden index, positive likelihood ratio, and negative likelihood ratio were calculated.

Our sample size fulfilled the minimum requirement recommended by the CLSI guidelines (120 observations for cut-off validation and 100 samples for assay comparison) [24, 25]. For the prognosis prediction equivalence assessment, the sample size was thought to have approximately 95% power (1-β) to detect a difference between the two assays with a 0.05 two-tailed significance level [28]. All statistical analyses were conducted using MedCalc Statistical Software (version 20.027; MedCalc Software Ltd, Ostend, Belgium). Rounding rules were applied to summary statistics, and a two-tailed *p*-value less than 0.05 was considered statistically significant [29].

## 3. Results

### 3.1 Validation of Clinical Cut-Off

Table 2 shows the clinical cut-off validation of Presage ST2, AFIAS ST2, and ichroma ST2 results. In both sample subsets, the 95th percentile URLs of AFIAS ST2 and ichroma ST2 were within the clinical cut-off of 35 ng/mL established using Presage ST2. There was no visible trend according to the assay or the origin of the samples.

**Table 2.** **Cut-off validation of Presage ST2, AFIAS ST2, and ichroma ST2 assay results**.

	95th percentile URL (ng/mL, 90% CI)		
Study population	Presage ST2	AFIAS ST2	ichroma ST2
AACC sample bank (n = 298)	29.79 (28.26–31.10)	33.22 (28.09–39.40)	32.98 (29.68–36.07)
KUMC healthy individuals (n = 122)	33.94 (29.91–38.04)	31.10 (27.83–39.31)	29.72 (27.73–40.05)

Abbreviations: AACC, American Association of Clinical Chemistry; CI, confidence interval; KUMC, Konkuk University Medical Center; n, number; URL, upper reference limit; ST2, suppression of tumorigenicity 2.

### 3.2 Comparison of Assay Results

**Fig. 1.**
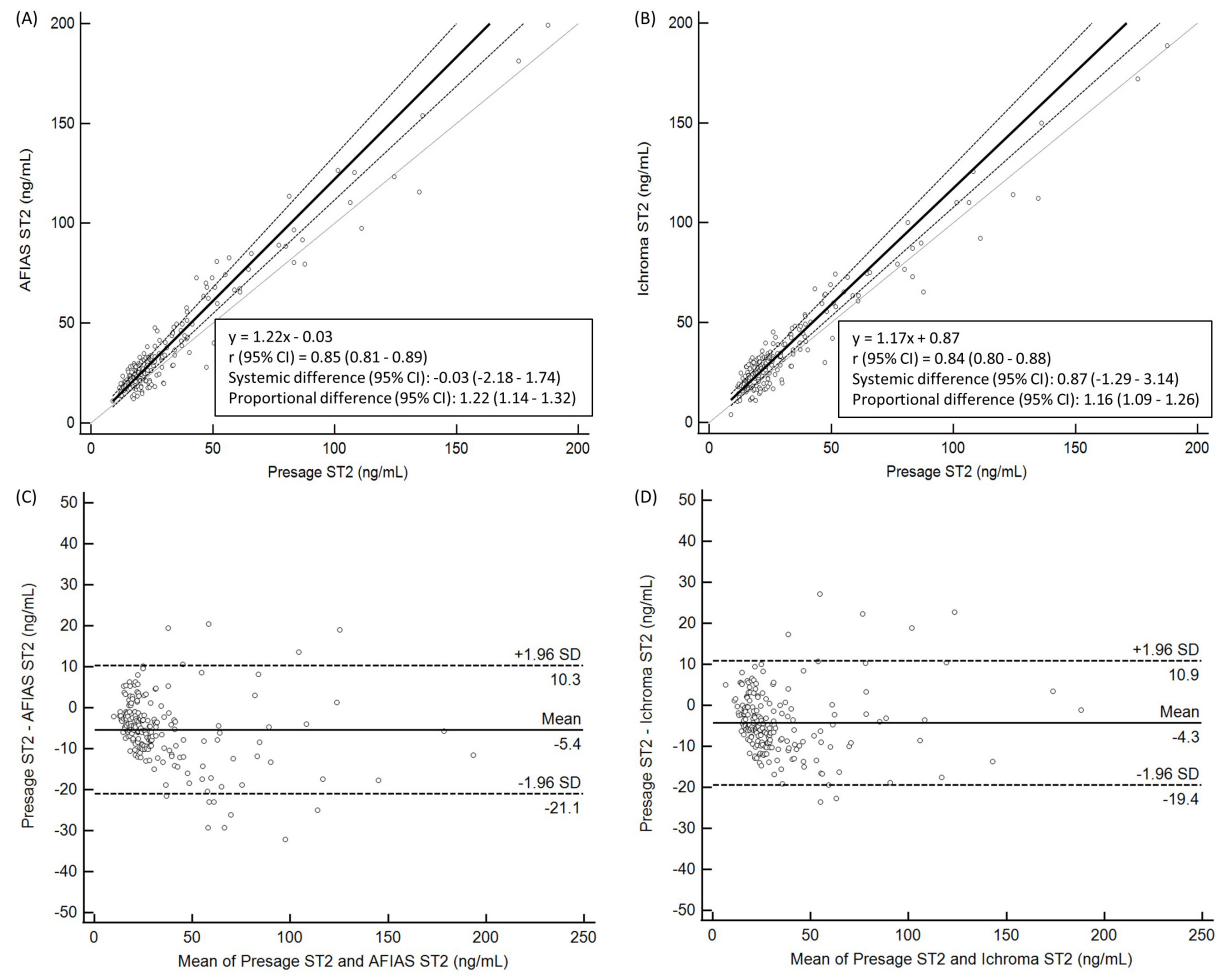
**Comparison of AFIAS ST2, ichroma ST2, and Presage ST2 assays using KUMC HF samples (n = 206)**. Correlation between (A) AFIAS ST2 vs. Presage ST2 and (B) ichroma ST2 vs. Presage ST2 using Passing–Bablok regression analysis. Differences between (C) AFIAS ST2 vs. Presage ST2 and (D) ichroma ST2 vs. Presage ST2 using Bland–Altman plots. All *p*-values were <0.001. Solid line, Passing–Bablok regression or mean difference; dashed line, 95% CI or ± 1.96 SD. Abbreviations: CI, confidence interval; HF, heart failure; KUMC, Konkuk University Medical Center; n, number; SD, standard deviation; ST2, suppression of tumorigenicity 2.

For the KUMC HF samples, both AFIAS ST2 and ichroma ST2 results were highly correlated with Presage ST2 results (r = 0.82 and 0.81, respectively); however, the former showed a positive proportional bias (+21% and +17%, respectively). The mean differences of AFIAS ST2 and ichroma ST2 with Presage ST2 were –4.8 ng/mL and –3.7 ng/mL, respectively (Fig. 1). The results of AFIAS ST2 and ichroma ST2 showed strong agreement with those of Presage ST2 (κ = 0.84 and 0.82, Table 3). The ST2 levels measured using AFIAS ST2 and ichroma ST2 were higher than those measured using Presage ST2.

**Table 3.** **Agreements between AFIAS ST2 and ichroma ST2 results and Presage ST2 results at the clinical cut-off in the KUMC HF samples (n = 206)**.

		Presage ST2		Weighted κ (95% CI)
		<35 ng/mL (n = 157)	≥35 ng/mL (n = 49)	
AFIAS ST2	<35 ng/mL (n = 146)	145	1	0.84 (0.75–0.92)
	≥35 ng/mL (n = 60)	12	48	
ichroma ST2	<35 ng/mL (n = 144)	143	1	0.82 (0.73–0.90)
	≥35 ng/mL (n = 62)	14	48	

Abbreviations: CI, confidence interval; HF, heart failure; KUMC, Konkuk University Medical Center; n, number; ST2, suppression of tumorigenicity 2.

### 3.3 Equivalence of Prognosis Prediction

**Fig. 2.**
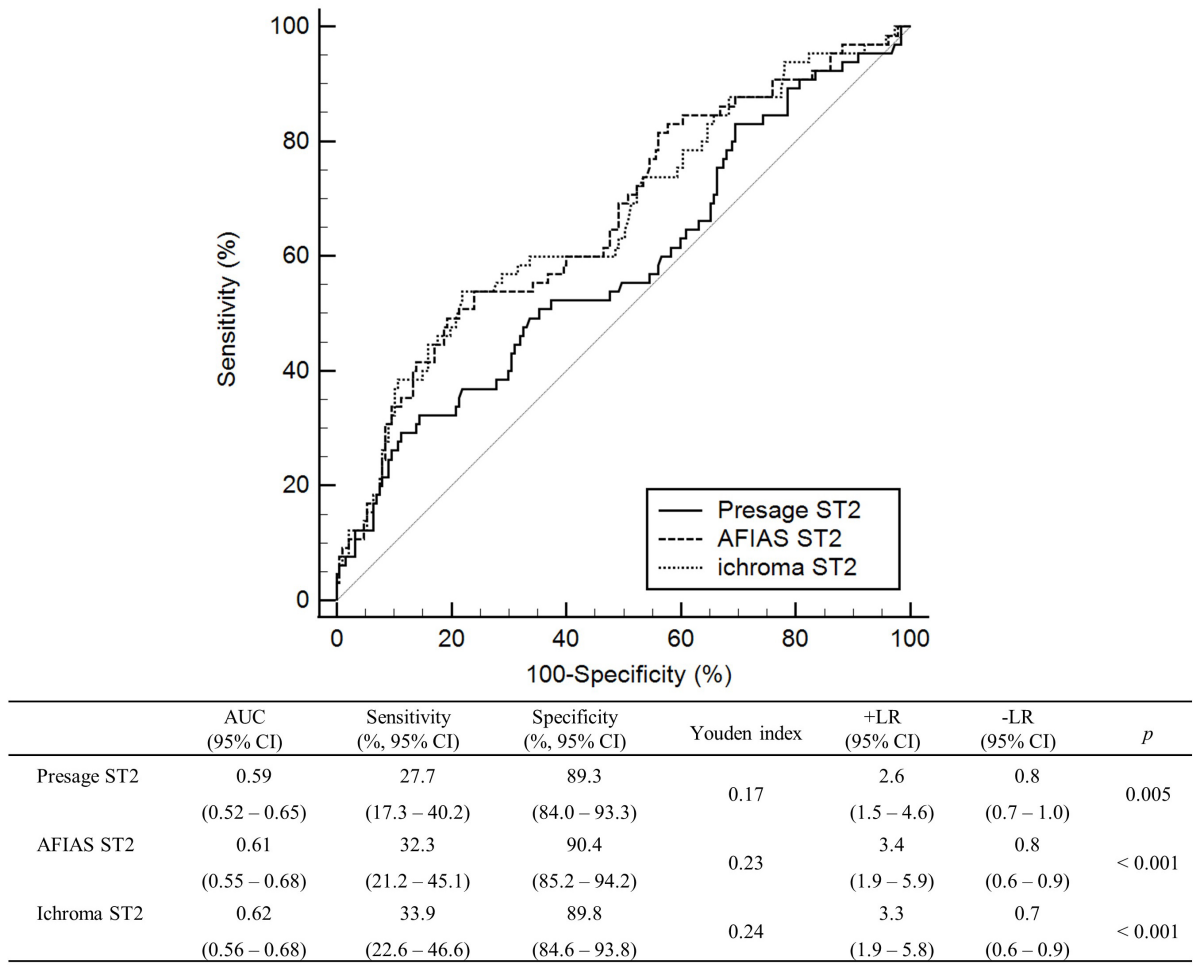
**ROC curve analyses of AFIAS ST2, ichroma ST2, and Presage ST2 assay results to predict CV death in the Barcelona ambulatory HF cohort subsets at a cut-off of 35 ng/mL (n = 252)**. Abbreviations: AUC, area under the curve; CI, confidence interval; CV, cardiovascular; HF, heart failure; n, number; ROC, receiver operating characteristic; ST2, suppression of tumorigenicity 2; +LR, positive likelihood ratio; -LR, negative likelihood ratio.

At a cut-off of 35 ng/mL, Presage ST2, AFIAS ST2, and ichroma ST2 could predict CV death in the Barcelona ambulatory HF cohort subsets; all three assays showed comparable AUCs, and there were no statistically significant differences across the results of the three assays (Fig. 2). In all three assays, the specificity for CV death was >85%.

## 4. Discussion

To the best of our knowledge, this is the first study to evaluate the performance of two newly developed automated fluorescence immunoassay-based POC sST2 assays: AFIAS ST2 and ichroma ST2. We compared the performance of these assays with that of ELISA-based Presage ST2 in terms of clinical cut-off, analytical performance, and prognosis.

The US FDA-approved clinical cut-off of 35 ng/mL was used based on the HF-ACTION Study using the Presage ST2 [11, 15, 16]. This cut-off is indicated for use in conjunction with clinical evaluation to assess the prognosis of HF. The present study showed that the URL results of AFIAS ST2 and ichroma ST2 were within the currently acknowledged clinical cut-off for Presage ST2 (Table 2). The AACC and KUMC samples yielded similar URL results in all three assays, suggesting that Presage ST2, AFIAS ST2, and ichroma ST2 are all applicable across races and ethnicities. In general, many biomarkers, including cardiac biomarkers, exhibit different reference intervals or clinical cut-offs depending on sex, race, and ethnicity [30, 31, 32, 33]. Based on a multi-ethnic, population-based cohort study of residents in Dallas County, African-American origin women and men had higher sST2 levels than Caucasian women and men [33]. Therefore, more rigorous validation of the sST2 clinical cut-off is needed considering ethnicity and sex in the participants regardless of the assay differences.

Various ST2 assays exhibited high proportional differences and were not directly comparable [12, 34]. The Aspect-PLUS ST2 could be compared with Presage ST2 but exhibited a positive proportional difference (+50%) [13]. Sequent-IA ST2 has been compared with Aspect-PLUS ST2 but not with Presage ST2 [14]. However, a clinical cut-off of 35 ng/mL was not validated in these studies [13, 14]. In the present study, AFIAS ST2 and ichroma ST2 results were highly correlated and exhibited positive proportional bias with Presage ST2 results (+21% and +17%, respectively) (Fig. 1). However, both AFIAS ST2 and ichroma ST2 exhibited acceptable agreement with Presage ST2 based on a clinical cut-off of 35 ng/mL (Table 3).

Including the HF-ACTION study, previous studies showed that elevated sST2 levels, higher than 35 ng/mL, predicted all-cause mortality, all-cause hospitalization, CV death, and CV hospitalization [6, 11, 35, 36, 37, 38]. Therefore, the FDA approved Presage ST2 to assess HF patients’ prognosis [11]. This study demonstrated that AFIAS ST2 and ichroma ST2 are equivalent to the US FDA-approved Presage ST2 in predicting CV death (Fig. 2).

### 4.1 Limitations

This study had several limitations. First, we compared the three sST2 assays using 206 samples from patients with HF. Due to the relatively small sample size and lack of serial samples from the same patients, in-depth analyses were not conducted considering HF classification and the number of recurrent hospitalizations. Second, we analyzed 252 samples from the Barcelona ambulatory HF cohort to predict CV death. Further large-scale studies are needed to determine the prognosis of HF among patients with elevated sST2 level in the Korean population. Third, we focused on validation of the clinical cut-off, comparison of analytical performances, and assessment of equivalence for prognosis prediction among the three assays. Due to limited clinical and laboratory information on the AACC sample bank and Barcelona ambulatory HF cohort, we could not include other demographic and baseline data, such as body mass index, waist circumference, and lipid profiles. Further, we could not analyze the prognostic significance considering the sST2 levels using the three ST2 assays and HF classification by left ventricular ejection fraction according to the AHA/ACC/HFSA guidelines [1]. Further studies are needed to explore prognosis through these ST2 assays, depending on the HF classification according to the AHA/ACC/HFSA guidelines. Finally, we did not analyze the turnaround time (TAT) or hands-on time for each POC assay. Based on the manufacturer-claimed TAT, we determined that AFIAS ST2 and ichroma ST2 are more suitable for clinical practice than ELISA.

### 4.2 Future Directions

Considering the increasing prevalence of preclinical stages of HF, early diagnosis and personalized POC strategies are required for HF management [6]. AFIAS and ichroma ST2 are newly launched IVD POC assays and could be an easy-to-use option for sST2 level measurement. Using these POC assays, clinicians can make immediate clinical decisions when treating HF patients, which may decrease the overall medical burden. Further research is required regarding the relationship between decreased TAT, a shorter hospitalization period, and medical cost reduction in the real world.

## 5. Conclusions

This study demonstrated that AFIAS ST2 and ichroma ST2 were equivalent to Presage ST2 in terms of clinical cut-off, assay performance, and prognosis prediction. AFIAS ST2 and ichroma ST2 are new easy-to-use POC assays for measuring sST2 levels in clinical practice.

## Data Availability

The datasets used and/or analyzed during the current study are available from the corresponding author on reasonable request.

## Abbreviations

AACC, American Association for Clinical Chemistry; ACC/AHA, American College of Cardiology/American Heart Association; BNP, B-type natriuretic peptide; CI, confidence interval; CLSI, Clinical & Laboratory Standards Institute; CV, cardiovascular; CV%, coefficient of variation; ELISA, enzyme-linked immunosorbent assay; FDA, Food and Drug Administration; HbA1c, hemoglobin A1c; HDL-C, high-density lipoprotein cholesterol; HF, heart failure; HF-ACTION study, HF: A Controlled Trial Investigating Outcomes of Exercise Training study; IL, interleukin; IQR, interquartile range; KUMC, Konkuk University Medical Center; LDL-C, low-density lipoprotein cholesterol; LoD, limit of detection; LoQ, limit of quantification; LVEF, left ventricular ejection fraction; NT-proBNP, N-terminal-prohormone B-type natriuretic peptide; NYHA, New York Heart Association; POC, point-of-care; ROC, receiver operating characteristic; SD, standard deviation; sST2, soluble suppression of tumorigenicity 2; URL, upper reference limit; yrs, years; +LR, positive likelihood ratio; LR, negative likelihood ratio.
